# Anxiety and depression during pregnancy in Central America: a cross-sectional study among pregnant women in the developing country Nicaragua

**DOI:** 10.1186/s12888-015-0671-y

**Published:** 2015-11-17

**Authors:** T. Verbeek, R. Arjadi, J. J. Vendrik, H. Burger, M. Y. Berger

**Affiliations:** Department of Epidemiology, University of Groningen, University Medical Center Groningen, HPC FA40, Postbus 30.001, 9700 RB Groningen, The Netherlands; Department of General Practice, University of Groningen, University Medical Center Groningen, Groningen, The Netherlands; Department of Clinical Psychology and Experimental Psychopathology, University of Groningen, Groningen, The Netherlands

**Keywords:** Pregnancy, Depression, Anxiety, Nicaragua, Central America

## Abstract

**Background:**

Around the world, maternal psychopathology during pregnancy is associated with a range of negative consequences for mother and child. Nevertheless, in Central America the magnitude of this public health problem is still unknown. The objective of this first explorative study was to investigate the prevalence and severity of anxiety and depression during pregnancy in the Central American developing country Nicaragua, as well as the availability of mental health care and to compare with a developed country.

**Methods:**

A population-based cohort of pregnant women in Nicaragua (*N* = 98) was compared with a parallel cohort in the Netherlands (*N* = 4725) on symptoms of anxiety (Spielberger State Trait Anxiety Inventory) and depression (Edinburgh Postnatal Depression Scale). Associations with the women’s knowledge how to reach professional psychological support were assessed using multivariable linear regression analyses.

**Results:**

Of the Nicaraguan women, 41 % had symptoms of anxiety and 57 % symptoms of depression, versus 15 % and 6 % of the Dutch women. Symptom scores of both anxiety and depression were significantly higher in Nicaragua (*p* < 0.001). However, only 9.6 % of the women indicated that professional psychological help was available for the Nicaraguan pregnant women, which was associated with an increased anxiety score.

**Conclusions:**

In Nicaragua, both prevalence and severity of symptoms of antenatal anxiety and depression are substantially higher than in developed countries. However, availability of psychological help is very limited for pregnant Nicaraguan women. These findings indicate that there is need for further research and support for these women, to prevent negative consequences for both mother and child.

## Background

According to the World Health Organization (WHO), mental, neurological, and substance abuse (MNS) disorders are responsible for 14 % of the global burden of disease in both men and women [1]. Especially during pregnancy, anxiety and depression are highly prevalent and are known to have a range of serious negative consequences for the child [2–6]. Experiencing symptoms of anxiety and depression during pregnancy is the most crucial risk factor for having these symptoms in the postpartum period [6, 7]. In developed countries, this has been associated with insecure mother-child attachment [8], preterm birth, low birth weight [9, 10], and emotional, cognitive, and behavioral problems in the offspring [6, 11]. Ultimately, psychosocial complaints in pregnancy have also been associated with increased maternal mortality [2, 6].

For the developed world, the occurrence of anxiety and depression during pregnancy has been intensively researched. Prevalence studies in developed countries show a prevalence of 10–15 % for depression and/or anxiety during pregnancy [6, 9, 12, 13]. Furthermore, research in developed countries shows that anxiety and depression are highly comorbid [9].

In developing countries, mental health has not received much attention from the research community. Depression and anxiety rates among pregnant women are less well known, but presumably higher. Indeed, it has been estimated that in developing countries, one-in-three to one-in five pregnant women experiences a significant mental health problem, comparing to one-in-ten in developed countries [4]. It is assumed that this high prevalence is the result of lower socioeconomic development of the population, abuse, violence and deficiency in mental health care [4]. The latter is confirmed in the 2011 WHO Mental Health Atlas, which showed that only 36 % of people living in low-income countries are covered by mental health care [14].

Nicaragua is the largest country in Central America, with a population of over six million people and a nominal gross domestic product of $2006 per capita [15]. Unemployment rate is high, more than 30 % of the population has a purchasing power parity of only $2 per day or less, women are low educated, and the majority of the women is illiterate [16]. Although the economical situation seems to improve over the years [15, 16], in healthcare there are still several worrying issues. For example, Nicaragua is one of the few countries in the world that forbid all abortions, even when a woman’s life is in danger [17]. In case this may daunt a pregnant woman: access to mental healthcare is limited, with only 2.92 psychiatrists and psychologists per 100,000 inhabitants, compared to 33.82 per 100,000 in the European developed country the Netherlands [14].

To our knowledge, the prevalence of depression and anxiety during pregnancy and their comorbidity are not known for the Central America region. We hypothesize that the prevalence and severity of both anxiety and depression are higher in developing countries than in developed countries. This study aims to explore the occurrence of these common forms of psychopathology among pregnant women in a low-income country in this region, particularly Nicaragua.

In this explorative study, we investigated differences in prevalence and severity of anxiety and depression symptoms in pregnant women as assessed in two parallel population based cohorts between Nicaragua and the Netherlands. Additionally, we investigated the correlations between depression and anxiety levels in pregnant women. Furthermore, we discussed the women’s knowledge how to reach mental health professionals and we examined whether this knowledge is associated to the severity of depression and/or anxiety symptoms.

## Methods

### Study design and participants

The present analyses were carried out using two population-based cohorts: a Nicaraguan cohort and the Pregnancy, Anxiety and Depression (PAD) Study in the Netherlands.

The Nicaraguan cohort consisted of pregnant women who visited the Luis Felipe Moncada public hospital in San Carlos or the community health centers (Centros de Saludes) in San Carlos and Los Chiles, two villages in the rural south of Nicaragua. Women visited the hospital or the community health center for regular pregnancy consultations or in the final phase of pregnancy. During the inclusion period, July – September 2013, all pregnant women were invited to participate in this cross-sectional study, in which all data were collected anonymously and without follow-up. All 105 women visiting the hospital (*N* = 44, 41.9 %) or the participating community health centers (*N* = 61, 58.1 %) were asked to join the study, of which 98 were willing to participate (93 %). When the participant was a minor, we obtained informed parental consent for them to participate. When women were not able to read the questionnaire due to illiteracy (*n* = 54), the researchers or nurses read the questionnaire aloud. We believed this was a better method then excluding all unlettered women. The only women who were excluded from participation were women who had no oral mastery of the Spanish language (*N* = 0). Written informed consent was obtained from each participant. This study was approved by dr. F. Ruiz and dr. M. Romero, directors of both the hospital and the community health centers, on behalf of the medical ethical review board of the Nicaraguan Ministry of Health, Managua, Nicaragua.

The PAD-study is an ongoing prospective cohort study in the Netherlands which has been set up to investigate symptoms of and risk factors for anxiety or depression during and after pregnancy [18]. As in the Nicaraguan cohort, all pregnant women in their first trimester of pregnancy visiting one of the 109 collaborating primary obstetric care centers and nine hospitals in the Netherlands were invited to participate. The only women who were excluded from participation were women who had shown no mastery of the Dutch language. Non-participation and exclusion were not systematically registered in the PAD-study. Nevertheless, a survey among participating midwives and gynecologists indicated that the vast majority of them stated that pressure of time meant that they could not hand out the forms to all the visiting women and that they did not specifically invited women they suspected to have symptoms of depression or otherwise. Therefore, we have no reason to believe that responders and non-responders differed in any considerable way with respect to the characteristics relevant to the study [13]. Data used for the present cross-sectional analyses was collected from May 2010 to September 2013. By the end of that period 4725 women, of whom 4229 (89.5 %) were included in one of the participating midwifery practices and 496 (10.5 %) in one of the participating hospitals, had completed the first follow-up questionnaire at twenty-three weeks GA, including anxiety and depression questionnaires. The PAD-study was approved by the medical ethical review board of the University Medical Center Groningen, Groningen, the Netherlands.

### Measurements

Demographic and pregnancy related variables included in the present study were maternal age and gestational age.

The Spielberger State Trait Anxiety Inventory (STAI) was used to assess the level of anxiety. We used the six-item short-form, because this instrument produces scores similar to those obtained using the full-form, with a lower burden. The cut-off score for an at least moderate level of anxiety is >42 in this short-form [19]. This commonly used questionnaire has a good internal consistency (average Cronbach’s alpha of .89) [20].

The 10-item Edinburgh Postnatal Depression Scale (EPDS) was used to assess the level of depressive symptoms [21]. Although the EPDS was originally developed to assess postnatal depression, the questionnaire has demonstrated to reliably assess depressive symptoms during pregnancy as well [22]. The cut-off score for an at least moderate level of depression is ≥12. The 10-item EPDS has shown good internal reliability with a Cronbach’s Alpha of 0.82 [23].

In the Nicaraguan cohort, we assessed the women’s knowledge how to reach professional support for psychosocial problems (yes/no). We differentiated between support of doctors, nurses, social workers, and psychotherapists. Professional support for psychosocial problems is easily accessible, widely available, and covered by health insurances of practically everyone in the Netherlands. Therefore, it was not assessed in the PAD-study.

### Statistics

First, descriptive statistics for demographic and pregnancy related variables were calculated. Differences were tested using independent sample t-tests.

Second, the differences in symptom levels of both anxiety and depression between the Nicaraguan and the PAD cohort were assessed using Mann–Whitney U tests because their distributions were skewed. Furthermore we calculated the differences in proportions of women above the cut-off score for an at least moderate level of anxiety (STAI >42) and depression (EPDS ≥12) and tested these differences using Chi^2^ tests of independence.

Third, a Pearson correlation coefficient was calculated for symptoms of anxiety and depression, for both the Nicaraguan and the PAD cohort.

Finally, in the Nicaraguan cohort we performed logistic regression analyses to quantify the link between the women’s knowledge how to reach psychological help (dependent variable) and symptoms of anxiety and depression during pregnancy (independent variables). For reasons of interpretation we standardized the symptom scores for anxiety and depression to a standard normal distribution, i.e. we created Z-scores. Consequently, the odds ratios obtained from the logistic regression analyses denote the relative increase in the odds of the knowledge how to reach help per standard deviation of symptoms score. To obtain results that can be considered dimension specific, we performed additional analyses in which we adjusted the analyses of anxiety symptoms for the level of depressive symptoms by adding depression symptom level as independent variable, and vice versa. To correct for shared variance, we added maternal age and gestational age as independent variables. Hosmer and Lemeshow goodness-of-fit tests were performed as regression diagnostics, considering *p* > 0.05 as a good fit.

All analyses were performed with SPSS 22 (IBM, USA). The level of statistical significance was conventionally set at 0.05, two-sided. All data is available upon request.

## Results

As shown in table 1, at the time of completing the questionnaires, mean gestational age of women in the Nicaraguan cohort was approximately 7 weeks higher than in the PAD study. Mean maternal age of the Nicaraguan women was circa 7 years lower than in the Dutch cohort. Differences in mean gestational and maternal ages were statistically significant (*p* < 0.001).

**Table 1:** Descriptive statistics

	Nicaragua (*N* = 98)	Netherlands (*N* = 4,725)	
Mean maternal age, years (range, SD)	23.6 (13–43, 6.9)	30.7 (16–44, 4.5)	*p* < 0.001
Mean gestational age, weeks (range, SD)	30.6 (8–40, 9.5)	23.5 (18–27, 1.9)	*p* < 0.001
STAI-score, median	36.7	33.3	*p* < 0.001
STAI-score > 42, N (%)	40 (41)	699 (15)	*p* < 0.001
EPDS-score, median	13.0	4.0	*p* < 0.001
EPDS-score > 11, N (%)	56 (57)	280 (6)	*p* < 0.001

Symptom levels of anxiety and depression were assessed using STAI- (min-max = 20–80) and EPDS-questionnaires (min-max = 0–30) during pregnancy. Cut-off values STAI > 42 and EPDS > 11 indicate moderate symptom levels of anxiety and depression, respectively

Differences were tested using independent sample t-tests, Mann–Whitney U tests, and Chi^2^ tests of independence where appropriate

*EPDS* Edinburgh Postnatal Depression Scale, *SD* Standard deviation, *STAI* Spielberger State Trait Anxiety Inventory

Mean STAI score in the Nicaraguan cohort was 38.7 (median = 36.7, SD = 13.7, min = 20, max = 70) and in the PAD study 33.3 (median = 33.3, SD 9.7, min 20, max 80). Mann Whitney U, U = 174638, *n*_1_ = 98, *n*_2_ = 4617, *p* < 0.001. A total of 40 women (41 %) had a STAI score above 42 (indicating at least moderate symptoms of anxiety), compared to 699 (15 %) in the Netherlands (Chi^2^ test of independence *p* < 0.001).

Mean EPDS score of the pregnant women in Nicaragua was 12.3 (median = 13.0, SD = 5.5, min = 0, max = 25) and in the Netherlands 5.0 (median = 4.0, SD = 3.7, min = 0, max = 27). Mann Whitney U, U = 64506, *n*_1_ = 98, *n*_2_ = 4600, *p* < 0.001. A total of 56 women (57 %) had an EPDS score above 11 (indicating at least moderate symptoms of depression), compared to 280 (6 %) in the Netherlands (Chi^2^ test of independence *p* < 0.001).

The STAI- and EPDS-scores were moderately correlated (Pearson’s *r* = 0.33, 95 % CI = 0.14 – 0.52, *p* = 0.001) in Nicaragua and highly correlated in the Netherlands (Pearson’s *r* = 0.73, 95 % CI = 0.71 – 0.75, *p* < 0.001).

In Nicaragua, only 9 of the 94 (9.6 %) women who completed this question indicated that professional psychological help is available for them. The STAI score was significantly associated with the knowledge how to reach psychological support (OR = 1.361, 95%CI = 1.016 – 1.824, *p* = 0.039). Conversely, the EPDS score showed no statistically significant association with this knowledge (OR = 1.044, 95%CI = 0.785 – 1.045, *p* = 0.623). Adjustment of the analysis of anxiety symptoms for the level of depressive symptoms, and vice versa, as well as adding maternal age and gestational age did not notably affect the results. Finally, repeating all analyses in both illiterate and non-illiterate women showed similar results.

## Discussion

In this first explorative study assessing antenatal psychopathology in Central-America, we found higher prevalences of anxiety and depression than observed in developed countries. The scores in our Nicaraguan sample were substantially higher than in the Netherlands and this difference was statistically significant. Further, we found a weaker correlation between anxiety and depression scores in Nicaragua than in the Netherlands. Additionally, although there may be psychological help available for the Nicaraguan pregnant women, these women were mostly not aware of this possibility or they were unable to attain this help. We found a significant association between women’s knowledge how to reach psychological help for these women and symptoms of anxiety, but not with depression.

With respect to the burden imposed by mental disorders, mental health is known to be an under-researched health area, especially during pregnancy [24]. Although the WHO recognises psychopathology as an important global health problem which causes morbidity and mortality in both mother and child, the problem may be even bigger than earlier thought [1–4]. Most earlier published prevalences of depression and/or anxiety during pregnancy were found in developed countries and were lower compared to those we found in our Nicaraguan sample, namely 10-15 % [6, 9, 12].

Literature demonstrating prevalences of antenatal anxiety and depression in developing countries outside Central America showed various results. For example, in Nigeria a low prevalence of 10.5 % for generalized anxiety disorder was found among pregnant women [25], but the severity and effect of anxiety symptoms (e.g. worry, avoidance, and obsessions) may not always rise to the level of an anxiety disorder diagnosis [26]. In contrast, in Brazil, the prevalence rate of anxious symptoms among pregnant women is estimated to be 60 %, and the rate for depressive symptoms was about 20 % [27]. Furthermore, in Bangladesh the prevalence rate of depressive symptoms among pregnant women is estimated to be 33 % [28] and 42.7 % in Pakistan [29]. The prevalences of anxiety (41 %) and depression (57 %) in our sample of Nicaraguan pregnant women were comparable to these earlier studies in developing countries but notably higher than in developed countries.

Unlike in developed countries, where anxiety and depression are highly correlated [9], in our Nicaraguan sample the observed correlation was substantially lower. Although possibly (partly) caused by the limited sample size, this finding suggests that anxiety and depression may have different origins, more often so in developing countries.

Although we neither performed a longitudinal study nor we searched for causal factors, the results of this first study on this subject in Nicaragua, indicate the need for further research. Compared to developed countries, lower education, lower income, younger maternal age, and more negative or traumatic life events could be factors in Nicaragua that relate to a higher risk of suffering from psychopathology during pregnancy [4, 30].

As one of the low-income countries in the Central American region, Nicaragua reported only 1 % of the total health care budget is reserved for mental health, and 91 % of that is given to psychiatric hospitals [14]. Under this condition, it is likely that relatively mild mental health issues in a specific population like pregnant women are neglected.

A very small proportion of the women, less than ten percent, indicated that psychological help was available and that they knew how to reach that help. This statement was associated with a higher anxiety score, suggesting that the anxious women know how to find psychological help. This was not the case for the depressive women. In any case, for the women in the rural areas psychological help may not be commonly available, so besides more knowledge about the problems, the possibilities of providing effective treatment if needed, e.g. psychotherapy for antenatal psychopathology, should be explored. It would be desirable to investigate the results of this possible solution in a follow-up study in the same geographical area.

The present study is not without limitations. First, both the STAI as the EPDS are self-report questionnaires. Even though these are worldwide commonly used questionnaires, misunderstandings of the questionnaire, possibly due to illiteracy, may have led to over- or under-reporting. Earlier research showed that a lower educational level was associated with a higher rate of psychopathology during pregnancy [30]. Nevertheless, when a participating woman was unlettered, we read the questionnaire aloud. We believed this was a better method then excluding all illiterate women. Furthermore, our analyses showed similar results in both illiterate and non-illiterate women. Secondly, cut-off values for both STAI and EPDS questionnaires may depend on different cultural backgrounds. However, since this is the first study among Nicaraguan women, we considered it justified using the widely recognised cut-off values for an at least moderate level of anxiety (STAI >42) [19] and depression (EPDS ≥12) [22]. Third, the sample sizes differed strongly between both samples, diminishing statistical power. Nevertheless, we demonstrated statistically significant differences between the Dutch and Nicaraguan groups. Finally, in our longitudinal Dutch cohort, women received multiple questionnaires during pregnancy, including STAI and EPDS questionnaires at twenty-three weeks gestational age. Our Nicaraguan cohort consisted of women who participated at any moment of pregnancy, with a mean gestational age of 30.6 weeks. The mean gestational age may be comparable (end of second versus start of third trimester), but the SD was increased fivefold.

A strong point is in our view that we were able to include a population-based sample of Nicaraguan pregnant women in a rural area, from both a public hospital and community health centers and had a remarkably high response rate (93 %). Because of the use of equal questionnaires in both Nicaragua and a large sample in the Netherlands, we were able to compare symptomatology of antenatal psychopathology between Nicaragua and a developed country. This study only explored the presence of anxiety and depression and the women’s knowledge how to reach psychological help, but since this was the first study on antenatal psychopathology in a rural area in Central-America, this publication can be a supplement to the literature on this topic.

## Conclusions

In conclusion, this study suggests that antenatal anxiety and depression in Central-America are important public health problems. Both prevalence as severity of symptoms of anxiety and depression during pregnancy are higher than we knew from earlier research. This study indicates that there is need for further research and support for these Nicaraguan women.
